# Thyrotoxic Periodic Paralysis in a Young Hispanic Male With Newly Diagnosed Grave's Disease

**DOI:** 10.7759/cureus.15814

**Published:** 2021-06-21

**Authors:** Colten M Mabile, Kuroush Nezafati

**Affiliations:** 1 Internal Medicine, Methodist Health System, Dallas, USA

**Keywords:** thyrotoxic periodic paralysis, graves disease, hypokalemia, hyperthyroid, hypokalemic periodic paralysis

## Abstract

Thyrotoxic periodic paralysis (TPP) is a unique cause of hypokalemia from transcellular shift into muscle in the setting of active thyrotoxicosis. It is essential to recognize TPP, given the specific management considerations, which would otherwise easily go unaddressed. TPP can also be clinically indistinguishable from other causes of hypokalemia. In particular, familial periodic paralysis can present similar to TPP. This case illustrates a young Hispanic male who presented with paralysis and was found to be hypokalemic. Patient was also found to have thyromegaly with further testing consistent with Grave's disease, despite no hyperthyroid symptoms. Ultimately, identifying TPP early will allow for swift and appropriate treatment, avoid unnecessary interventions and testing, and reduce cost of care.

## Introduction

Hypokalemia is a potential life-threatening electrolyte disturbance, with complications resulting from muscle paralysis and dysfunction, including in cardiac muscle and potentially life-threatening arrhythmias [1]. Etiology of hypokalemia helps dictate the degree of potassium repletion and additional interventions. Thyrotoxic periodic paralysis (TPP) is a rare but an important cause of hypokalemia. The overall mechanism involves intracellular potassium shift from excess thyroid hormone. The illness may or may not present simultaneously with typical hyperthyroid symptoms. Definitive treatment requires eventual correction of hyperthyroidism. We present a case of TPP, along with discussion of management, to add to the literature and share awareness of this rare cause to a common internist problem.

## Case presentation

A 20-year-old Hispanic male with a past medical history of depression presents to the ED with a complaint of bilateral lower-extremity weakness and pain. He reports that the leg weakness and pain began about one week prior to presentation. On the day he presented to the ED, the lower-extremity weakness had worsened, preventing him from getting out of bed. This was the first time he experienced such weakness. He works as a landscaper and has been unable to work for the past week. He denies any trauma to his lower extremities or back, leg swelling, or leg rash. He does not take any medications at home. He reports using marijuana, but no recent use. He is adopted and is unaware of any family history.

**Figure 1:**
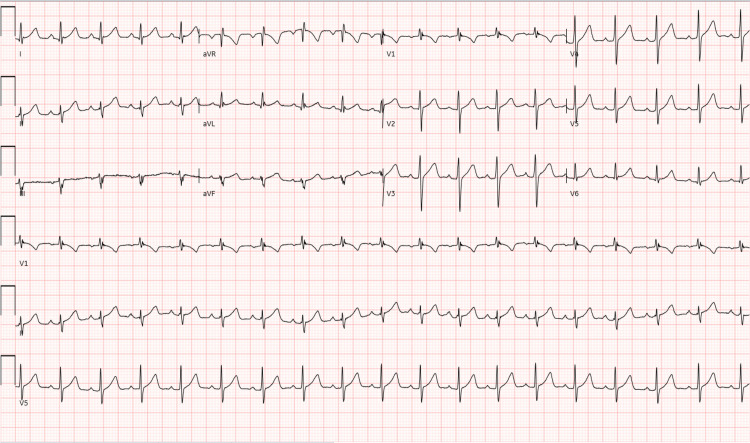
ECG with no significant abnormalities present

On presentation, patient was afebrile (body temperature 36.2°C), with a regular heart rate (82 beats per minute), and normotensive (131/77 mmHg). Physical examination was notable for intact cranial nerve exam, no nuchal rigidity, mild thyromegaly with no palpable nodules, 5/5 strength in left upper extremity, 2/5 strength throughout the right upper extremity, 2/5 strength throughout the left lower extremity, and 3/5 strength throughout the right lower extremity. Sensation was intact in all four extremities. Initial laboratory data were notable for a potassium of 2.1 mmol/L and magnesium 1.7 mg/dL. ECG revealed normal sinus rhythm and no ST depression or U waves (Figure 1).

**Figure 2:**
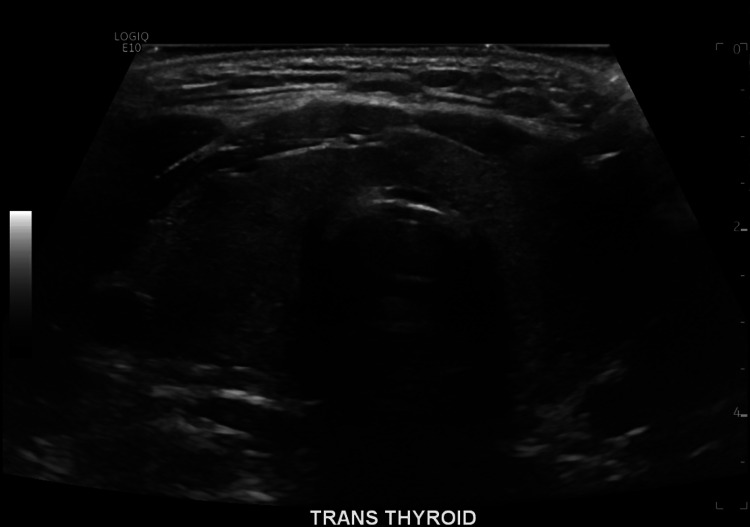
Ultrasound revealing an inhomogeneous, mildly enlarged thyroid

**Figure 3:**
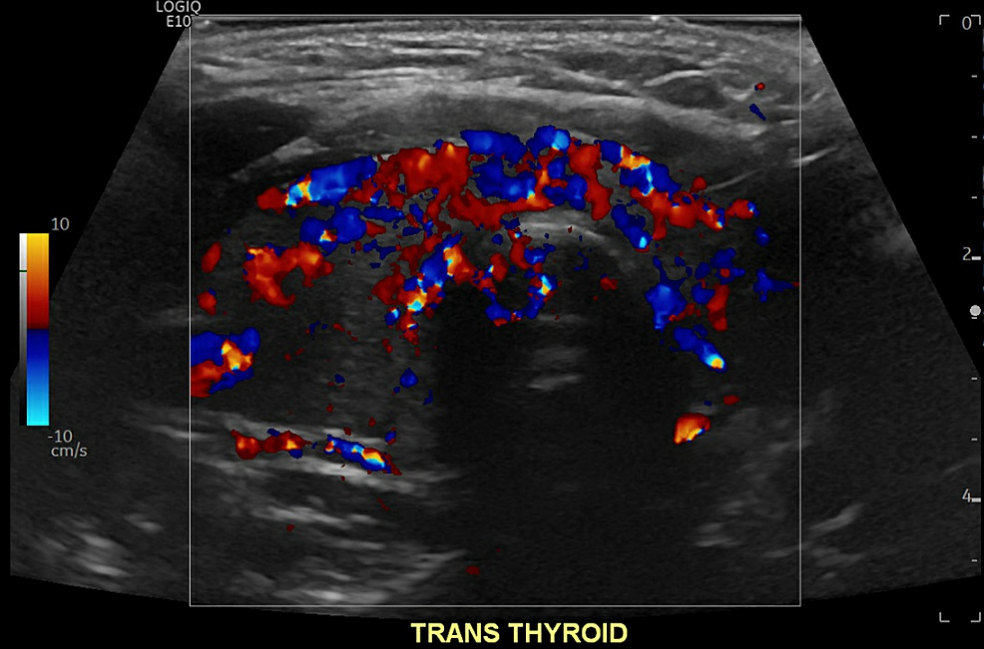
Thyroid Doppler with increased vascularity

**Figure 4:**
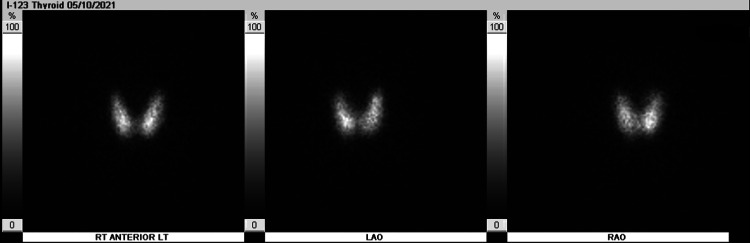
A radioactive iodine uptake scan was obtained showing uniform tracer uptake throughout the thyroid gland with 6-hour iodine uptake measuring 67% (normal 6-18%) and 24-hour iodine uptake measuring 51% (normal 10-35%)

Patient received 80 mEq of potassium chloride orally and 40 mEq intravenously. Altogether, these were administered to the patient over the course of 6 hours from presentation in the ER. Potassium on recheck corrected beyond the expected amount to 5.1 mmol/L. Patient’s weakness resolved while receiving the potassium. This was clinically suspicious for TPP. Therefore, thyroid values were checked, which showed TSH to be undetectably low at <0.02 uIU/mL, free T4 elevated at 4.62 ng/dL, and free T3 elevated significantly at 19.20 pg/mL. Due to the concern for thyrotoxicosis, patient was started on propranolol 30 mg daily. A thyroid ultrasound was obtained, showing a mildly enlarged and inhomogeneous thyroid gland with no nodules and hypervascularity on Doppler (Figures 2, 3). A thyroid uptake scan revealed increased uptake uniformly (Figure 4).

Thyroid-stimulating immunoglobulins were elevated at 8.14 IU/L. This clinical picture was consistent with Grave's disease. The final diagnosis was TPP secondary to Grave’s disease. Patient had left against medical advice prior to the results of the radioactive iodine uptake scan and thyroid-stimulating immunoglobins laboratory test coming back, and, therefore, had not been started on definitive treatment.

## Discussion

TPP is a rare type of acquired, sporadic hypokalemic periodic paralysis. TPP comprises 0.1-0.2% of thyrotoxic patients in North America, and 1.8-8.8% in Japan and 1.9% in China, respectively [2]. TPP has clinical features indistinguishable from familial periodic paralysis (FPP), an autosomal dominant hypokalemic periodic paralysis, which typically presents in younger individuals <20 years of age. Both TPP and FPP patients typically display proximal muscle weakness, cramping, aching, and even paralysis, with normal acid-base chemistry and mild hypophosphatemia [3]. Patient can even have absent reflexes consistent with generalized myopathic pathology. TPP is typically considered acquired in the setting of thyrotoxicosis that may or may not have symptoms on presentation. Pathogenesis is not fully understood but believed to be contributed by the increased expression and activity of Na+/K+-ATPase in skeletal muscle in the setting of increased thyroid hormone [3]. This activity can also be increased by insulin after a high-carbohydrate meal, or catecholamine release [4]. A large subset of TPP patients also have a mutation in a gene-expressing inward-rectifying potassium channel, Kir2.6. Kir2.6 is a channel expressed on skeletal muscles, with effects on membrane excitability and whose transcription is regulated by the thyroid hormone. Some mutations in Kir2.6 lead to effects on excitability and paralysis [5].

Understanding the pathophysiology of this rare cause of TPP is critical to the appropriate treatment, as the cause is entirely from transcellular shift. Current recommendations for potassium repletion in TPP reflect this pathophysiology. Treatment consists of a combination of a non-selective beta blocker such as propranolol, along with potassium repletion [6]. It is notable that repletion alone may not be sufficient to correct the potassium [7]. If potassium is given, it is to best to be given in small amounts until paralysis or weakness resolves. This is an effort to avoid rebound hyperkalemia, which has been known to occur in this patient population [8]. To avoid rebound hyperkalemia, potassium can be given at a rate below 10 mEq/hour, unless there is a cardiopulmonary complication [9]. This approach is keeping in mind that the hypokalemia is due to transcellular shift. 

Administering non-selective beta blockers such as propranolol will help reduce activity of the Na+/K+-ATPase, reduce insulin secretion, and correct the underlying cause, which is increased thyroid hormone (through reduction of T4 to T3) [10]. Dosing of propranolol can be 3 mg/kg PO, if patient can tolerate [11]. Otherwise, IV propranolol can be given 1 mg at a time every 10 minutes repeated up to 3 mg total [12].

## Conclusions

TPP is a rare and an important cause of hypokalemia. This case demonstrates the importance of a thorough evaluation of hypokalemia etiologies. The case also highlights the potential severity and treatment considerations, given its unique pathophysiology. Standard of care currently utilizes both non-selective beta blockers and potassium repletion until symptom resolution. In this case, patient was identified as having this entity early on, which allowed us to avoid unnecessary workup for this patient. Further, over-correction of potassium from return of potassium from intracellular shifts (a well-described phenomenon in TPP) was avoided by early suspicion of this disease. 
